# Prosaposin Reduces α-Synuclein in Cells and Saposin C Dislodges it from Glucosylceramide-enriched Lipid Membranes

**DOI:** 10.1007/s12031-022-02066-y

**Published:** 2022-09-24

**Authors:** Rika Kojima, Mark Zurbruegg, Tianyi Li, Wojciech Paslawski, Xiaoqun Zhang, Per Svenningsson

**Affiliations:** 1 Department of Clinical Neuroscience, Neuro Svenningsson, Karolinska Institutet, 171 76 Stockholm, Sweden; 2 Basic and Clinical Neuroscience, King’s College London, London, UK

**Keywords:** α-Synuclein, Autophagy, Glucocerebrosidase, Prosaposin, Saposin C

## Abstract

Parkinson’s disease (PD) is the second most common progressive neurodegenerative disorder affecting over 1% of the 65 + age population. Saposin C, a lysosomal protein required for the normal activity of glucocerebrosidase (GCase), may serve as a disease modifier in PD. Saposin C is cleaved from its precursor, Prosaposin (PSAP), which is secreted as an uncleaved protein and exerts neuroprotective effects. In this study, we aim to elucidate the neuroprotective roles of PSAP and saposin C in PD by evaluating their effects on α-synuclein accumulation in human neuroblastoma cells. Stable overexpression of PSAP reduced monomeric α-synuclein levels in SH-SY5Y cells, while PSAP knockdown by small interfering RNA led to the opposite effect, and those effects were independent of GCase activity. Autophagy flux was decreased by stable PSAP overexpression. Furthermore, a flow-through assay revealed that recombinant saposin C was able to detach α-synuclein from artificial glucosylceramide-enriched lipid membranes at the lysosomal pH. Taken together, our findings provide further evidence that PSAP and saposin C as key proteins involved in α-synuclein clearance by dislodging it from lipid membranes.

**Supplementary Information:**

The online version contains supplementary material available at 10.1007/s12031-022-02066-y.

## Introduction

Parkinson’s disease (PD) is the second most common progressive neurodegenerative disorder characterized by the loss of dopaminergic neurons in the substantia nigra (Poewe et al. 2017). The dopamine deficiency caused by the dopaminergic cell death results in motor symptoms particularly rest tremors, rigidity, and bradykinesia. Those symptoms can be counteracted by dopamine replacement therapies, especially in the early stages of the disease (Lees et al. 2009). However, the effect of the medication wears off as the disease progresses (Armstrong and Okun 2020). Although many patients are coping with the disease by controlling symptoms with the help of medications or deep brain stimulation, there are still no treatments that can slow down the progression of PD.

The causes of the specific neuronal cell death remain unknown, but misfolding and aggregation of α-synuclein are involved in PD pathology (Braak et al. 2003; Meade and Fairlie 2019). α-Synuclein is a major component of Lewy bodies, which are inclusion bodies found in affected neurons of PD patients (Lashuel et al. 2012). α-Synuclein is part of the SNARE complex and its normal function relates to neurotransmitter release (Burré et al. 2010) and some mutations or multiplications in the *SNCA* gene, which encodes α-synuclein, are known to cause PD (Polymeropoulos et al. 1997).

Mutations in the *GBA1* gene are the most common risk factor for developing PD (Do et al. 2019). The *GBA1* gene encodes glucocerebrosidase (GCase), a lysosomal protein which breaks down glucocerebroside into glucose and ceramide. More than 495 mutations in the *GBA1* gene are known to cause impairment in glycosphingolipid metabolism, and *GBA1* mutation homozygosity leads to Gaucher’s disease (GD) (Behl et al. 2021). Even asymptomatic heterozygote *GBA1* mutation carriers have a higher risk of developing PD, which is comparable to GD patients (Avenali et al. 2020). Despite the fact that their GCase activity is strongly reduced, only a smaller set of GD patients develop PD symptoms (Alcalay et al. 2014). Although reduced GCase activity is associated with both idiopathic and familial PD (Alcalay et al. 2015), it is still largely unknown how GCase contributes to the development of PD.

Prosaposin (PSAP) is a neurotrophic and neuroprotective factor that can be found in various body fluids such as milk and cerebrospinal fluid (Hineno et al. 1991; O’Brien et al. 1994; Sikora et al. 2007). In addition to its neuroprotective roles, PSAP is cleaved in the late endosomes/lysosomes and serves as a precursor of saposins A–D, which are essential activators for several lysosomal hydrolases (Kishimoto et al. 1992; Leonova et al. 1996). Complete loss of PSAP leads to a neonatally lethal phenotype in both mice and humans (Harzer et al. 1989; Fujita et al. 1996), indicating that PSAP plays an important role in normal cellular functions.

Among four saposins, saposin C, which is required for a normal function of GCase, has been suggested as a disease modifier of GD (Tamargo et al. 2012). Deficiency in saposin C is known to cause neuropathological types of GD (Vaccaro et al. 2010; Tamargo et al. 2012). Yap et al. have shown that saposin C protects GCase against α-synuclein inhibition by displacing α-synuclein from lipid membranes (Yap et al. 2013). Also, previous studies have reported that the neuroprotective effect of PSAP is attributed to a short amino acid sequence in the N-terminal part of the saposin C region (O’Brien et al. 1995; Gao et al. 2013). TX14(a), a 14-mer prosaptide derived from the neurotrophic region in the saposin C domain of PSAP has neuroprotective effects equivalent to full-length PSAP (Hiraiwa et al. 1997b; Tsuboi et al. 1998). Some studies argue that the neuroprotective effects of prosaptides are mediated by the orphan G-protein coupled receptors, GPR37 and GPR37L1, but no consensus has been reached on this argument (Meyer et al. 2013; Leinartaité and Svenningsson 2017; Giddens et al. 2017; Liu et al. 2018).

In this study, we showed that overexpression of PSAP reduced monomeric α-synuclein levels in SH-SY5Y cells while PSAP knockdown by siRNA lead to the opposite effect, and those effects were independent of GCase activity. Moreover, we found that recombinant saposin C was able to dislodge α-synuclein from the artificial glucosylceramide-enriched vesicles at the lysosomal pH 5.4. Our findings suggest that PSAP and saposin C work as neuroprotective factors at least partly by inhibiting α-synuclein aggregation via replacing it from the lysosomal membrane.

## Materials and Methods

### Chemicals

Unless stated otherwise, all chemicals were purchased from Sigma-Aldrich (Merck KGaA) and were of analytical grade. All solutions were made using Milli-Q deionized water (Millipore).

### Cell Culture

SH-SY5Y cells were purchased from American Type Culture Collection (CRL-2266™, 70019544) and grown in a 1:1 mixing of MEM (11095–080, Gibco) and F-12 K (21127–022, Gibco) containing 10% FBS (10500–064, Gibco), NEAA (11140–035, Gibco), Sodium Pyruvate (11360–039, Gibco), and Penicillin–Streptomycin (100U/mL, 15140–122, Gibco). Cells were split 1:5 every 3–5 days.

### Generation of Transgenic Cell Lines

PSAP-GFP (HG16224-ACG, Sino Biological) and control-EGFP (13031, Addgene) plasmids underwent amplification in *E. coli* and were purified using plasmid extraction kits (12143, Qiagen). Transfection into SH-SY5Y cells was done using Lipofectamine 2000 (11668019, Invitrogen) as per manufacturer instructions. PSAP-OE cells were selected by 200 μg/mL of Hygromycin B (10687010, Gibco) for 30 days. EGFP-OE cells were selected by 300 μg/mL of Geneticin (10131027, Gibco) for 30 days. Stable overexpression of EGFP or PSAP-GFP has been confirmed by Western blot and observation under a fluorescent microscope. Cells were subsequently kept in low concentration antibiotics (100 μg/mL) during experiments. GFP fluorescence was examined routinely during experiments to ensure consistent protein expression.

### GCase Activity Assay

Cells were washed with PBS and lysed in TNT buffer (100 mM Tris–HCl (pH 7.4), 100 mM NaCl, 0.2% Triton X-100) containing protease inhibitor (cOmplete™ Mini, 04693159001, Roche) for 30 min on ice. Cells were spun down at 16,000 × g for 10 min at 4 °C and supernatant was collected. Total protein content in lysates was determined by bichronic acid (BCA) assay (23225, Thermo Scientific). For non-specific GBA activity, 30 μg of the total protein was transferred into an Eppendorf tube and mixed with the activity assay buffer (Citrate–Phosphate buffer pH 5.4, 1% bovine serum albumin, 0.25% Triton X-100, 0.25% mM Sodium taurocholate, 0.1% EDTA), and 1 mM 4-methylumbelliferyl β-d-glucopyranoside (44059, Glycosynth). The Eppendorf tubes were inverted gently multiple times to ensure equal distribution of reagents and then incubated for 2 h at 37 °C. The reaction was terminated by the addition of 25% 1 M Glycine–NaOH buffer (pH 10.5). The reaction product was loaded into a black 96 well plate in duplicate. Fluorescence was measured on a Tecan Spark 10 M (Tecan, Switzerland) (Ex: 360 / Em: 449). For GCase activity, 30 μg of the total protein was mixed with the activity assay buffer and incubated with 5 nM AMP-dNM (10010332, Cayman Chemical) for 30 min at room temperature. Then, 1 mM 4-methylumbelliferyl β-d-glucopyranoside (M3633, Sigma-Aldrich) was added to the lysates. The reaction mixture was loaded into a black 96 well plate in duplicate and the fluorescence was measured on a Tecan Spark 10 M every 15 min for 9 h at 37 °C. GCase activity was determined by analyzing the relative slopes calculated from 0 to 6 h measurements.

### Live-cell Imaging with Confocal Microscopy

Cells were seeded on an 8 well-chambered coverglass (155411, Thermo Fisher Scientific) and imaged on a Carl Zeiss LSM 880 confocal microscope using a 40 × 1.4 NA oil immersion objective. Before the imaging was performed, cell culture media was replaced with phenol red-free media. 50 nM LysoTracker™ Deep Red (L12492, Invitrogen) was directly added to the cell culture media and incubated for 15–60 min before the image acquirement. For RAB5/RAB7 co-localization experiment, RAB5-RFP or RAB7-RFP plasmids (14437 and 14436, Addgene) were transfected to PSAP-OE cells 24 h before the experiment using Lipofectamine 2000 (11668019, Invitrogen) as per manufacturer instructions.

### Pharmacological Treatments

Pharmacological treatments were done by replacing media containing each drug. Conduritol B epoxide (CBE) (C5424, Sigma-Aldrich), Bafilomycin A1 (B1793, Sigma-Aldrich), or MG132 (1748, Tocris) was added to the cell culture media to the final concentration of 100 μM, 100 nM, or 10 μM, respectively. The identical volume of DMSO was added to the media for the vehicle control group. Cells were incubated in the cell culture media containing CBE, Bafilomycin A1, or MG132 for 5 days, 2 h, or 4 h, respectively. For CBE treatment, old cell culture media was replaced with fresh media containing CBE after 72 h. After the treatment, cells were collected and subjected to Western blot and/or GCase activity assay.

### siRNA Transfection

*PSAP* siRNA (s529240, Thermo Fisher Scientific), and Negative control siRNA, (AM4611, Thermo Fisher Scientific) were diluted in Opti-MEM (11058021, Thermo Fisher Scientific) and mixed with Lipofectamine RNAiMax (13778030, Thermo Fisher Scientific). The mixture was mixed by tapping and incubated for 5 min at room temperature. Old media was removed and fresh media containing 10 nM siRNA was added. Cells were cultured for 10 days. Cells were split upon confluence. New siRNA/Lipofectamine was added every 72 or 96 h. After 10 days, cells were collected and subjected to Western blot and real-time PCR analyses.

### Western Blot

Cell lysates were extracted in RIPA buffer (150 mM sodium chloride, 1.0% Triton X-100, 0.5% sodium deoxycholate, 0.1% SDS, 50 mM Tris, pH 8.0) containing protease inhibitor (cOmplete™ Mini, 04693159001, Roche). The protein content of samples was determined by BCA assay (23225, Thermo Fisher Scientific). Samples were diluted to equal protein concentrations in lysis buffer and mixed with 4 × SDS-loading buffer (1610747, Bio-rad). 10 or 20 µg of the sample was loaded into each well. The semi-dry transfer was done at 2.5 A for 11 min onto the PVDF membrane (1704275, Bio-Rad). Membranes containing α-synuclein were placed in 4% PFA, 0.02% glutaraldehyde for 30 min and subsequently washed 3 times with TBS-T. Membranes were then blocked in 5% bovine serum albumin (A9647, Sigma-Aldrich) or Intercept™ (TBS) Blocking buffer (927–60001, LI-COR). Primary antibodies were applied overnight at 4 °C, except for β-actin which was applied for 2 h at room temperature. Antibodies used in the study are indicated in Table 1.

**Table 1:** Antibodies used for Western blot

**Antibody**	**Host**	**Company**	**Cat. #**	**Dilution factor**
**Primary antibodies**				
Anti-α-synuclein (C20)	Rabbit	SantaCruz	sc-7011-R	1:2000
Anti-PSAP	Rabbit	Atlas antibodies	HPA004426	1:1000
Anti-GCase	Rabbit	Abcam	Ab128879	1:1000
Anti-LC3b	Rabbit	Cell Signaling	2775S	1:1000
Anti-SQSTM1/p62	Rabbit	Cell Signaling	5114S	1:1000
Anti-β-actin	Mouse	Sigma-Aldrich	A2228	1:10,000
Anti-GAPDH (6C5)	Mouse	SantaCruz	sc-32233	1:1000
Anti-GFP	Rabbit	Abcam	AB6556	1:3000
**Secondary antibodies**				
IRDye 800CW Goat Anti-Rabbit IgG	Goat	Licor	P/N 926–32211	1:20,000
IRDye 680RD Goat Anti-Mouse IgG	Goat	Licor	P/N 926–68070	1:20,000

The signals from target proteins were read on LI-COR Odyssey CLx (LI-COR Biosciences) or ChemiDoc™ MP Imaging System (Bio-rad). Western blot quantification was done by Image Studio ver 5.2. Obtained signals from each target protein were normalized to the signals from the housekeeping proteins (β-actin or GAPDH) in the same lane. Relative signal intensities represented in the percentage of the control group were calculated by each membrane. Then, the obtained relative signal intensities from each replicated experiment were integrated and statistically analyzed using GraphPad Prism v9.00, 7.03 or 5.04. The original whole blots are in Supplementary Fig. 1.

**Fig. 1:**
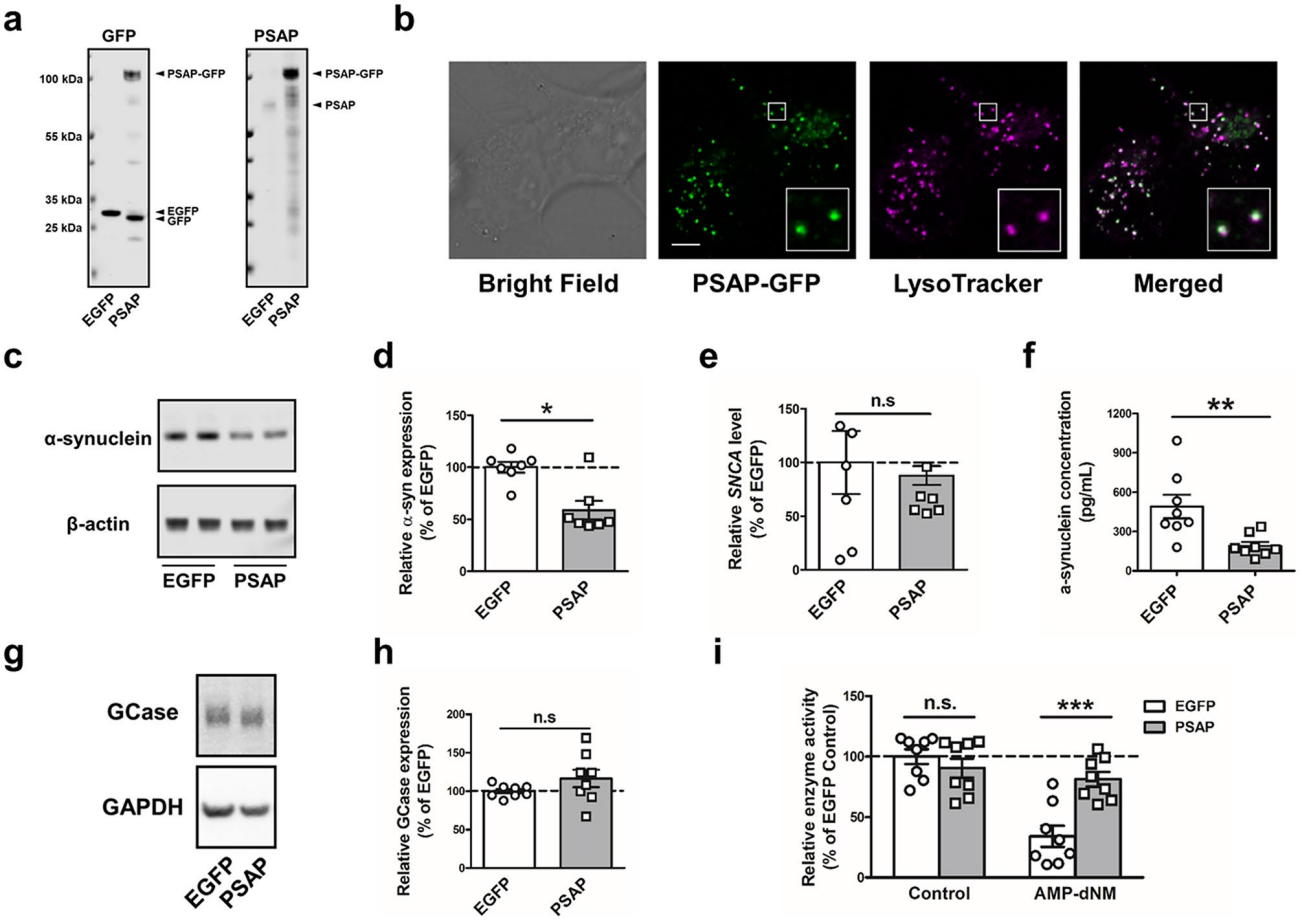
Overexpression of Prosaposin (PSAP) lowers levels of α-synuclein. (**a**) Representative Western Blot for anti-GFP (Left) and anti-PSAP (Right) in EGFP-Overexpressing (EGFP-OE) cells and PSAP-Overexpressing (PSAP-OE) cells showing PSAP-GFP at 105 kDa, endogenous PSAP at 70 kDa, and control EGFP at 32 kDa. (**b**) Representative live-cell images of PSAP-OE cells for PSAP-GFP co-localized with the lysosome (Green: PSAP-GFP, Magenta: LysoTracker, scale bar 5 µm). (**c**) Representative Western Blot for anti-α-synuclein and anti-β-actin in EGFP-OE cells and PSAP-OE cells. (**d**) Quantification of Western Blot analysis showing levels of α-synuclein in EGFP-OE cells (n = 10) and PSAP-OE cells (n = 8). (**e**) Real-time PCR analysis showing mRNA levels of *SNCA* in EGFP-OE cells (n = 6) and PSAP-OE cells (n = 6). (**f**) Quantification of α-synuclein levels in cell culture media of EGFP-OE cells (n = 8) and PSAP-OE cells (n = 8) measured by enzyme-linked immunosorbent assay. (**g**) Representative Western blot for anti-Glucocerebrosidase (GCase) and anti-GAPDH in EGFP-OE cells and PSAP-OE cells. (**h**) Quantification of Western blot analysis showing levels of GCase in EGFP-OE cells (n = 8) and PSAP-OE cells (n = 8). (**i**) Non-specific GBA activity and specific GCase activity analysis in EGFP-OE cells (n = 8) and PSAP-OE cells (n = 8) treated with or without AMP-deoxynojirimycin. Data are expressed as mean ± SEM. n.s: non-significant, *p* > 0.05; *p < 0.05; **p < 0.01; ***p < 0.001, Mann–Whitney *U* test (d, e, f, h,) and Two-way ANOVA with Tukey’s multiple comparison test (i)

### Real-time PCR

RNA was extracted with RNeasy Plus Mini Kit (74134, Qiagen). RNA concentration and quality were determined using Spark 10 M with a NanoQuant inset. QuantiTect Reverse Transcription Kit (205311, Qiagen) was used for cDNA synthesis. Real-time PCR was performed using Sso Advanced Universal SYBR green supermix (1725272, Bio-rad) with 2.5–5 ng of cDNA per reaction. *hRPL19* was used as an internal control to which the relative expression of *SNCA* and *PSAP* mRNA was normalized. The following primers were used: *hRPL19* 5′-ATGTATCACAGCCTGTACCTG-3′- and 5′–TTCTTGGTCTCTTCCTCCTTG-3′-, *hSNCA* 5′–ACCAAACAGGGTGTGGCAGAAG-3′- and 5′–CTTGCTCTTTGGTCTTCTCAGCC-3′-, *hPSAP* 5′–CCCGGTCCTTGGACTGAAAG-3′- and 5′–TATGTCGCAGGGAAGGGATTT-3′-. The reaction was initiated at 95 °C for 30 s and then 39 cycles of 95 °C for 15 s followed by 60 °C for 30 s. Melt curve analysis was performed to ensure the specificity of amplicons. Ct-values were converted to RNA expression levels using the 2^−ΔΔCt^ method.

### α-Synuclein ELISA

Extracellular α-synuclein concentration was measured using the Human α-Synuclein ELISA Kit (#844101, BioLegend) by following the manufacturer’s instructions. Obtained α-synuclein concentrations from 200 µL of undiluted cell culture media were normalized to total protein concentration from cells. For sample collection, cells were seeded on a 24 well plate and grown at 80–90% confluence. Then, the medium was replaced with 1 mL/well of the phenol-free medium and incubated for 48 h. The medium was transferred to an Eppendorf tube and centrifuged at 1500 rpm at 4 °C for 10 min. The supernatant was collected and stored at − 80 °C until use. After the medium collection, cells were lysed with RIPA buffer and total protein was determined by BCA assay for normalization.

### Generation of Glucosylceramide Lipid Vesicles

Vesicles were generated as previously described (Taguchi et al. 2017). Shortly, glucosylceramide and phosphocholine lipids were obtained from Avanti polar lipids (131304P, 840051P, Avanti Polar Lipids). Lipids were dissolved in a 2:1 chloroform/methanol mixture and dried in a glass vial under a nitrogen stream. Dried lipids were rehydrated in either 0.1 M phosphate buffer (pH 7.4) or citrate phosphate buffer (pH 5.4) for 1 h at room temperature to an end concentration of 138 μM with 25:75% experimental glucosylceramide: phosphocholine ratio. Vials were briefly vortexed and placed in an ultrasonic cleaning bath for 30 min. The lipids underwent two freeze/thaw cycles and were placed back in the bath for another 60 min. Lipids were extruded immediately to generate 100 nm diameter vesicles using the Avanti lipid extruder (610023, Avanti Polar Lipids).

### Generation of Recombinant Α-synuclein and Saposin C

α-Synuclein was prepared as previously described (Paslawski et al. 2016). Saposin C vector pET16b (Ahn et al. 2006), containing the insert coding for human saposin C, was expressed in *E. coli* Rosetta™ 2 (DE3) competent cells using an auto-induction method. The cells’ pellet was collected by centrifugation, suspended in the osmotic shock buffer (20 mM Tris–HCl, pH 7.2, 40% sucrose), incubated for 10 min and centrifuged again. Next, cells were treated with ice-cold deionized water, supplemented with saturated MgCl_2_ solution and briefly incubated on ice. The soluble fraction was collected, and the majority of proteins were depleted by adjusting pH to 3.5 with 1 M HCl. The remaining supernatant was collected by centrifugation and pH was adjusted to 7.5 with 1 M NaOH. The solution was filtered and fractionated on a Q-Sepharose column. Fractions containing saposin C were identified by SDS-PAGE and/or Western Blot, pooled together, dialyzed against deionized water and lyophilized. Obtained protein powder was suspended in 1 × PBS and fractionated using Superose 6 size exclusion column connected to the ÄKTA Explorer system (GE Healthcare). Afterwards, fractions containing saposin C were identified by SDS-PAGE and/or Western Blot, pooled together, dialyzed against deionized water and concentration was determined using NanoDrop ND-1000 (Thermo Fisher Scientific). Finally, the protein was aliquoted, lyophilized and stored at − 20 °C.

### Flow-through Assay

Fresh lipid vesicles were transferred to a clear 96 well plate and incubated in the presence of 5 μM recombinant α-synuclein and 5 μM experimental protein. Short synthetic saposin C peptides (TX14(a)) were obtained from Synpeptide and their purity and identity were verified by mass-spectrometry (Shanghai, China). Plates were incubated at 37 °C, with 95% humidity, 5% CO_2_ for 2 h. Incubated vesicles were loaded onto Ultracel 100 kDa spin filters (UFC510096, Merck-Millipore). Samples were spun at 14,000 × g for 30 min. Spin filters were washed by the addition of extra buffer followed by a 10-min spin at 14,000 × g. Spin filters were inverted in a new collection tube and the retained fraction was collected by spinning at 1000 × g for 2 min. Retained and flow-through fractions are mixed with 5 × loading buffer, heated to 95 °C immediately after spinning and subjected to Western blot analysis.

### Statistics

GraphPad Prism v9.00, 7.03 or 5.04 was used for the generation of all graphs and statistical analysis. Error bars represent the SEM. Student’s *t*-test or Mann–Whitney *U* test was used for pairwise comparisons depending on the normality of distribution. One-way ANOVA with Dunnett’s multiple comparison test or two-way ANOVA with Tukey’s multiple comparison test was used to compare multiple variables. Significance was set as follows: *p < 0.05; **p < 0.01; ***p < 0.001.

## Results

### Overexpression of PSAP Lowers Levels of Α-synuclein

To investigate the effect of PSAP expression on α-synuclein accumulation, we generated SH-SY5Y cells stably overexpressing PSAP (PSAP-OE cells). We used the plasmid containing green fluorescent protein (GFP)–linked PSAP, which allows us to monitor intercellular trafficking of exogenous PSAP. As a control, SH-SY5Y cells stably overexpressing enhanced-GFP (EGFP-OE cells) were also created. Overexpression of PSAP-GFP or EGFP from the plasmids was confirmed by Western blot (Fig. 1a). To check whether the exogenous PSAP-GFP is properly trafficked in the cells, live-cell imaging was performed. The lysosome was stained with LysoTracker, a fluorescent dye for labeling and trafficking the lysosome in live cells (Fig. 1b). Co-localization of PSAP-GFP and LysoTracker was observed in most of the cells, which indicates proper targeting of the lysosome. In addition, we also detected that a part of PSAP-GFP was co-localized with RAB5 and RAB7, which are early and late endosome marker proteins, respectively (Chavrier et al. 1990), suggesting that the exogenous PSAP was secreted into the cell culture medium and taken up by the neighboring cells (Supplementary Fig. 2).

**Fig. 2:**
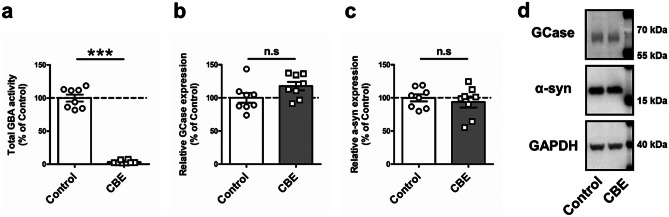
The decrease in levels of α-synuclein is GCase activity independent. (**a**) Non-specific GBA activity assay in PSAP-OE cells with or without CBE treatment (n = 8). (**b**) Quantification of Western blot analysis showing levels of GCase in PSAP-OE cells with or without CBE treatment (n = 8). (**c**) Quantification of Western Blot analysis showing levels of α-synuclein in PSAP-OE cells with or without CBE treatment (n = 8). (**d**) Representative Western blot for anti-GCase, anti-α-synuclein and anti-GAPDH in PSAP-OE cells with or without CBE treatment. Data are expressed as mean ± SEM. n.s: non-significant, ***p < 0.001, Mann–Whitney *U* test (a–c)

Next, α-synuclein levels in PSAP-OE and EGFP-OE cells were examined by Western blot. We showed that monomeric α-synuclein levels were significantly decreased in PSAP-OE cells compared with control EGFP-OE cells (Fig. 1c, d; Mann–Whitney *U* test: *p* = 0.0175). On the other hand, mRNA levels of *SNCA* were not affected by PSAP overexpression, suggesting that PSAP decrease the amount of α-synuclein at protein levels independent of gene transcription (Fig. 1e; Mann–Whitney *U* test: *p* = 0.6991). To examine α-synuclein secretion in EGFP-OE and PSAP-OE cells, α-synuclein levels in the cell culture medium were analyzed by enzyme-linked immunosorbent assay (ELISA). Extracellular α-synuclein was significantly decreased in PSAP-OE cells (Fig. 1f, Mann–Whitney *U* test: *p* = 0.0011). Thus, we showed that overexpression of PSAP lowers both intra- and extracellular α-synuclein levels without changing mRNA levels.

As Saposin C is known to enhance GCase activity (Weiler et al. 1993), we demonstrated the effects of PSAP overexpression on GCase expression and activity. The expression levels of GCase were not changed by PSAP overexpression (Fig. 1g, h; Mann–Whitney *U* test: *p* = 0.2345). Then, we examined non-specific GBA activity and specific GCase activity by treating the cells with 5 nM AMP-deoxynojirimycin (AMP-dNM), a specific GBA2 inhibitor (Overkleeft et al. 1998; Schöndorf et al. 2014) (Fig. 1i; two-way ANOVA with Tukey’s multiple comparison test: EGFP Control vs. PSAP Control: *p* = 0.7938; EGFP Control vs. EGFP AMP-dNM: *p* < 0.0001; PSAP Control vs. PSAP AMP-dNM: *p* = 0.8055; EGFP AMP-dNM vs. PSAP AMP-dNM: *p* = 0.0005).

GCase activity, represented as AMP-dNM unaffected fraction of the non-specific GBA activity, was 34% in EGFP-OE cells and 90% in PSAP-OE cells. Considering that PSAP overexpression does not alter GCase levels and non-specific GBA activity, this result suggests that PSAP may be able to compensate for disrupted GBA2 enzyme activity by promoting GCase activity.

### The Decrease in Levels of Α-synuclein is GCase Activity Independent

To investigate whether GCase activity is accounting for the reduction of α-synuclein levels in PSAP-OE cells, we used Conduritol B epoxide (CBE), an irreversible inhibitor of GCase, to suppress GCase activity in the cells. PSAP-OE cells were treated with CBE for 5 days. We confirmed that 100 μM CBE treatment potently decreased non-specific GBA activity (Fig. 2a; Mann–Whitney *U* test: *p* = 0.0002) without changing the levels of GCase (Fig. 2b, d; Mann–Whitney *U* test: *p* = 0.0650). We found no alteration of α-synuclein levels by CBE despite the robust GCase inhibition in PSAP-OE cells (Fig. 2c, d; Mann–Whitney *U* test: *p* = 0.7209), which is consistent with a previous report (Dermentzaki et al. 2013).

### Overexpression of PSAP Affects Autophagy

**Fig. 3:**
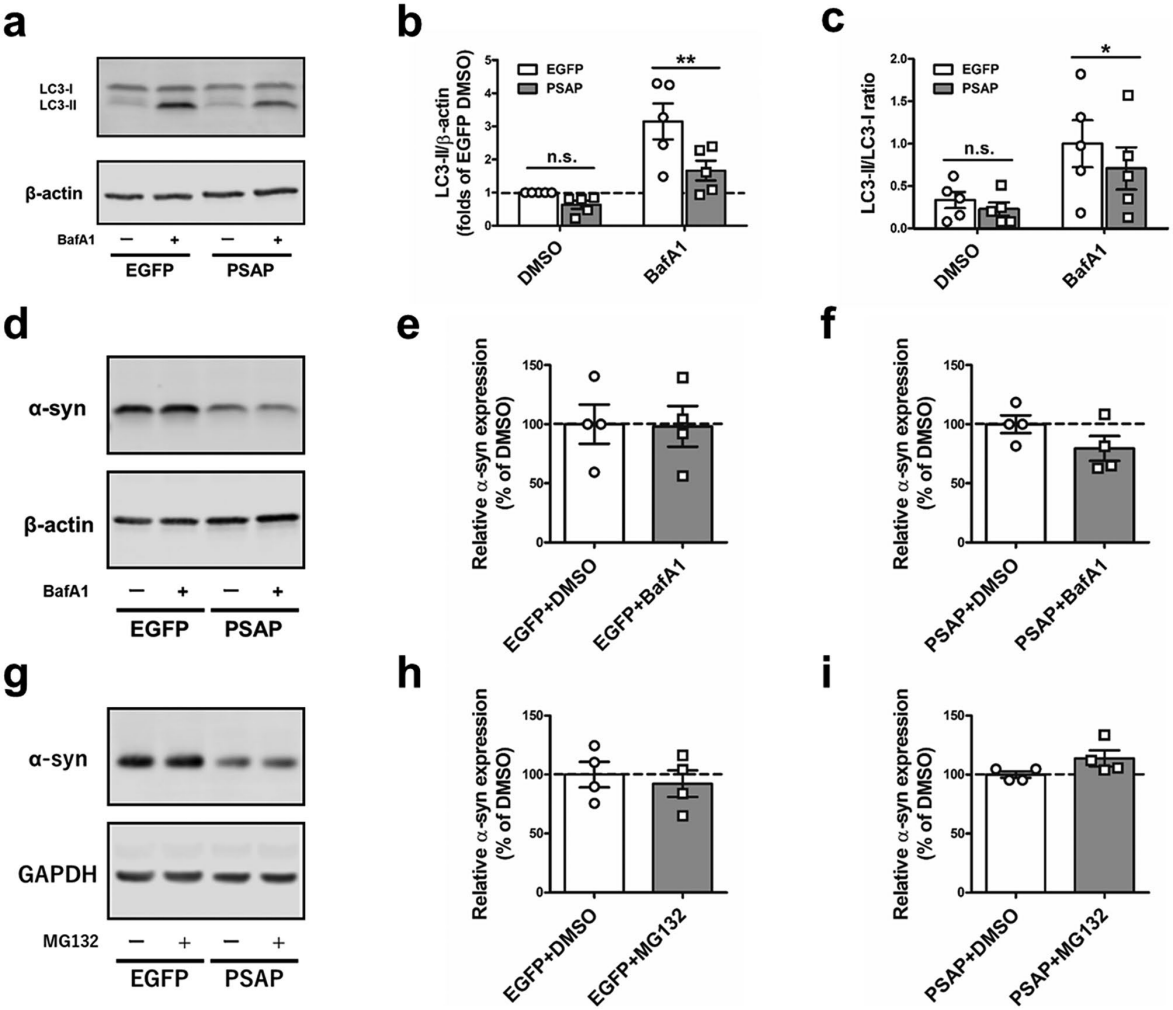
Overexpression of PSAP affects autophagy. (**a**) Representative Western blot for anti-LC3b and anti-β-actin in 100 nM Bafilomycin A1 or dimethyl sulfoxide (DMSO) treated EGFP-OE or PSAP-OE cells. (**b**) Quantification of Western blot analysis showing levels of LC3-II in 100 nM Bafilomycin A1 or DMSO treated EGFP-OE or PSAP-OE cells (n = 5). (**c**) Quantification of Western blot analysis showing LC3-II/LC3-I ratio in 100 nM Bafilomycin A1 or DMSO treated EGFP-OE or PSAP-OE cells (n = 5). (**d**) Representative Western blot for anti-α-synuclein and anti-β-actin in 100 nM Bafilomycin A1 or DMSO treated EGFP-OE or PSAP-OE cells. (**e**) Quantification of Western blot analysis showing levels of α-synuclein in 100 nM Bafilomycin A1 or DMSO treated EGFP-OE cells (n = 4). (**f**) Quantification of Western blot analysis showing levels of α-synuclein in 100 nM Bafilomycin A1 or DMSO treated PSAP-OE cells (n = 4). (**g**) Representative Western blot for anti-α-synuclein and anti-GAPDH in 10 µM MG132 or DMSO treated EGFP-OE or PSAP-OE cells. (**h**) Quantification of Western blot analysis showing levels of α-synuclein in 10 µM MG132 or DMSO treated EGFP-OE cells (n = 4). (**i**) Quantification of Western blot analysis showing levels of α-synuclein in 10 µM MG132 or DMSO treated PSAP-OE cells (n = 4). Data are expressed as mean ± SEM. *p < 0.05; **p < 0.01, Two-way ANOVA with Tukey’s multiple comparison test (b, c) and unpaired t-test (e, f, h, i)

Previous reports claimed that PSAP and saposin C deficiency increases autophagy (Vaccaro et al. 2010; Motta et al. 2016). To investigate whether PSAP overexpression has effects on autophagy, we checked the levels of LC3-I and LC3-II, common autophagosome markers to monitor autophagy flux, under basal and autophagy inhibition conditions (Tanida et al. 2008). EGFP-OE and PSAP-OE cells were incubated for 2 h with dimethyl sulfoxide (DMSO) or 100 nM Bafilomycin A1, a V-ATPase inhibitor blocking autophagosome-lysosome fusion and lysosomal acidification (Mauvezin and Neufeld 2015). Bafilomycin A1 treatment increased LC3-II levels in both cell lines, but the upregulation of autophagy was significantly weaker in PSAP-OE cells (Fig. 3a, b; two-way ANOVA with Tukey’s multiple comparison test: EGFP DMSO vs. PSAP DMSO: *p* = 0.2896; EGFP Bafilomycin A1 vs. PSAP Bafilomycin A1: *p* = 0.0036.) compared with EGFP-OE cells. The ratio of LC3-II/LC3-I, which is also commonly used as an indicator of autophagy flux, showed the same tendency (Fig. 3a, c; two-way ANOVA with Tukey’s multiple comparison test: EGFP DMSO vs. PSAP DMSO: *p* = 0.2621; EGFP Bafilomycin A1 vs. PSAP Bafilomycin A1: *p* = 0.0130.). We examined the levels of p62, a ubiquitin-binding protein which serves as another autophagy marker (Supplementary Fig. 3). There were no significant changes in p62 levels between EGFP- and PSAP-OE cells under basal or autophagy inhibition conditions. Also, p62 levels were not affected by Bafilomycin A1 treatment in either cell line. While LC3-II levels were substantially affected in PSAP-OE cells, the effect of PSAP overexpression on p62 levels was barely detected. This could be due to the nature of the complex regulations of p62 levels compared with LC3, which was argued in previous publications (Liu et al. 2016; Klionsky et al. 2021). To sum, overexpression of PSAP alters autophagy in the opposite direction of what has been reported in PSAP deficient cells.

Based on our observation that PSAP overexpression decreased both intracellular and extracellular α-synuclein without affecting mRNA levels, we considered that degradation of α-synuclein is upregulated in PSAP-OE cells. Since α-synuclein is known to be degraded by both autophagy and the ubiquitin–proteasome system, we investigated the degradation of α-synuclein in both pathways. First, to examine autophagy-lysosome-dependent degradation of α-synuclein, cells were treated with Bafilomycin A1 and α-synuclein levels were quantified by Western blot (Fig. 3d–f; Unpaired Student’s *t*-test: (Fig. 3e); F(6) = 0.08068, *p* = 0.9383 and (Fig. 3f); F(6) = 1.590, *p* = 0.1630). While LC3 accumulation was observed in Fig. 3a, the levels of α-synuclein were not changed in EGFP-OE and PSAP-OE cells. Next, to investigate the ubiquitin–proteasome–dependent degradation of α-synuclein, the cells were treated with MG132, a reversible proteasomal inhibitor, and α-synuclein levels were quantified by Western blot (Fig. 3g–i; Unpaired Student’s *t*-test: (Fig. 3h); F(6) = 0.4943, *p* = 0.6387 and (Fig. 3i); F(6) = 1.880, *p* = 0.1092). Similar to Bafilomycin A1 treatment, MG132 treatment did not affect α-synuclein degradation. These results indicate that the reduced levels of α-synuclein in PSAP-OE cells were not caused by direct upregulation of autophagy or ubiquitin–proteasome system.

### Saposin C Can Detach Α-synuclein From Glucosylceramide-enriched Lipid VesiclesiRNA Knock-down of PSAP Increases Levels of Α-synuclein

**Fig. 4:**
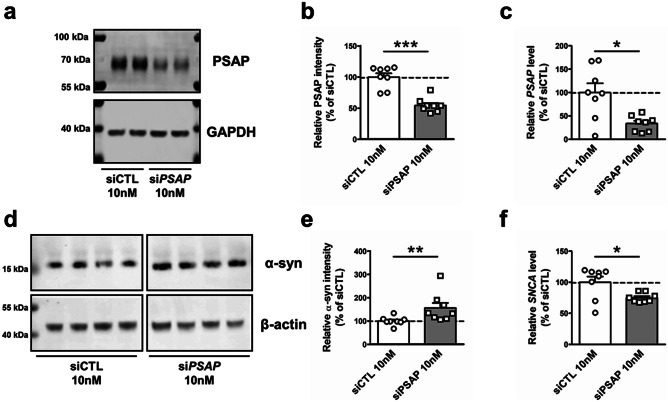
siRNA knockdown of *PSAP* increases levels of α-synuclein. (**a**) Representative Western blot for anti-PSAP and anti-β-actin in 10 nM siCTL or si*PSAP* treated WT SH-SY5Y cells. Endogenous PSAP is detected as an intracellular form of 68 kDa and deglycosylated form of ~ 50 kDa (Kishimoto et al. 1992). (**b**) Quantification of Western blot analysis showing levels of PSAP in siCTL or si*PSAP* treated WT SH-SY5Y cells (n = 8). (**c**) Real-time PCR analysis showing mRNA levels of *PSAP* in siCTL or si*PSAP* treated WT SH-SY5Y cells (n = 8). (**d**) Representative Western blot for anti-α-synuclein and anti-β-actin in siCTL or si*PSAP* treated WT SH-SY5Y cells. (**e**) Quantification of Western blot analysis showing levels of α-synuclein in siCTL or si*PSAP* treated WT SH-SY5Y cells (n = 8). (**f**) Real-time PCR analysis showing mRNA levels of *SNCA* in siCTL or si*PSAP* treated WT SH-SY5Y cells (n = 8). Data are expressed as mean ± SEM. n.s: non-significant, *p* > 0.05; *p < 0.05; **p < 0.01; ***p < 0.001, Mann–Whitney *U* test (b, c, e, f)

Given the result that the overexpression of PSAP reduced α-synuclein levels, we hypothesized that a decrease in PSAP levels would result in increased levels of α-synuclein. To test this hypothesis, we used siRNA targeting *PSAP* mRNA. First, we optimized siRNA concentration by using the non-specific GBA activity assay as a readout (Supplementary Fig. 4). *PSAP* knockdown caused a dose-dependent decrease in non-specific GBA activity. We decided to take 10 nM as the highest concentration of siRNA that did not show visible negative effects on cell growth while still decreasing GBA activity. WT SH-SY5Y cells were treated over 10 days with three subsequent 10 nM control siRNA or si*PSAP* transfections. Western blot confirmed reduced expression of PSAP in si*PSAP* treated cells (Fig. 4a, b; Mann–Whitney *U* test: *p* = 0.0006). Knockdown of *PSAP* was also validated at mRNA levels (Fig. 4c; Mann–Whitney *U* test: *p* = 0.0207). As we hypothesized, PSAP knockdown significantly increased α-synuclein levels (Fig. 4d, e; Mann–Whitney *U* test: *p* = 0.0047). However, mRNA levels of *SNCA* were decreased in si*PSAP*-treated cells, indicating the existence of feedback inhibition of *SNCA* transcription (Fig. 4f; Mann–Whitney *U* test: *p* = 0.0499).

### Saposin C Can Detach Α-synuclein From Glucosylceramide-enriched Lipid Vesicles

**Fig. 5:**
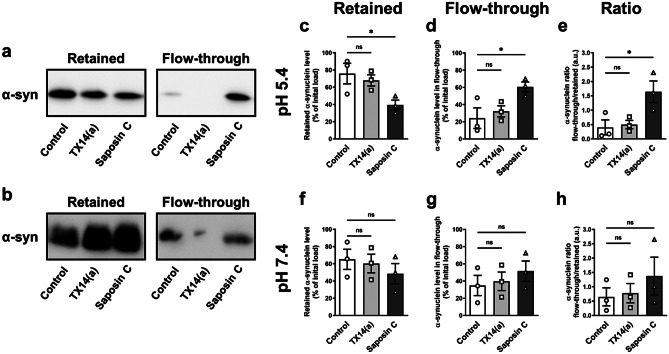
Saposin C can detach α-synuclein from glucosylceramide-enriched lipid vesicles. (**a**, **b**) Representative Western blot for anti-α-synuclein in following treatment of the vesicles; incubated with recombinant α-synuclein and control peptide (Control) or short synthetic saposin C peptide (TX14(a)) or recombinant saposin C (Saposin C) in either pH 5.4 or 7.4 buffer. After incubation, vesicles were separated into retained and flow-through fractions by centrifugation. Left blots show α-synuclein retained with vesicles and right blots show dislodged or unbound α-synuclein detected in flow-through. (**c**–**e**) Quantification of Western blot analysis from 3 independent experiments showing the amount of α-synuclein in retained fraction (**c**), flow-through fraction (**d**) and the ratio of flow-through over-retained α-synuclein (**e**) at lysosomal pH (pH 5.4). (**f**–**h**) Quantification of Western blot analysis from 3 independent experiments showing the amount of α-synuclein in retained fraction (**f**), flow-through fraction (**g**) and the ratio of flow-through over-retained α-synuclein (**h**) at cytoplasmic pH (pH 7.4). Data are expressed as mean ± SEM. n.s: non-significant, *p* > 0.05; *p < 0.05, n = 3 in each group, One-way ANOVA with Dunnett's multiple comparison test (c–h)

Based on previous reports showing that saposin C can interrupt GCase-α-synuclein interactions (Yap et al. 2013; Gruschus et al. 2015), we hypothesized that there is a direct competition between saposin C and α-synuclein. To test the hypothesis, we created artificial lipid vesicles and incubated them with recombinant α-synuclein and saposin C to examine how they influence each other’s levels in vitro. The vesicles consisted of glucosylceramide and phosphocholine, and either in a pH 7.4 phosphate buffer or a pH 5.4 citrate–phosphate buffer, which are cytoplasmic pH or lysosomal pH, respectively. Addition of recombinant saposin C, but not a control peptide or the neuroprotective prosaptide (TX14(a)), caused α-synuclein to be dislodged from the artificial vesicles at lysosomal pH 5.4 (Fig. 5a, c–e; One-way ANOVA with Dunnett’s multiple comparison test: (Fig. 5c); F (2, 6) = 5.076, *p* = 0.0513; Control vs. TX14(a): *p* = 0.7465; Control vs. Saposin C: *p* = 0.0405. (Fig. 5d); F (2, 6) = 5.076, *p* = 0.0513; Control vs. TX14(a): *p* = 0.7465; Control vs. Saposin C: *p* = 0.0405. (Fig. 5e); F (2, 6) = 6.203, *p* = 0.0346; Control vs. TX14(a): *p* = 0.9550; Control vs. Saposin C: *p* = 0.0340), but not at cytoplasmic pH 7.4 (Fig. 5b, f–h; One-way ANOVA with Dunnett’s multiple comparison test: (Fig. 5f); F (2, 6) = 0.5646, *p* = 0.5961; Control vs. TX14(a): *p* = 0.9372; Control vs. Saposin C: *p* = 0.5196. (Fig. 5g); F (2, 6) = 0.5646, *p* = 0.5961; Control vs. TX14(a): *p* = 0.9372; Control vs. Saposin C: *p* = 0.5196. (Fig. 5h); F (2, 6) = 0.7046, *p* = 0.5311; Control vs. TX14(a): *p* = 0.9716; Control vs. Saposin C: *p* = 0.4755). This result indicates that saposin C prevents α-synuclein from binding to glucosylceramide-enriched lipid membranes.

## Discussion

Our study shows that PSAP regulates the levels of α-synuclein, which is attributed to saposin C’s function to promote α-synuclein clearance by dissociating α-synuclein from the lipid membranes. We demonstrate that α-synuclein levels are decreased/increased by PSAP overexpression/knockdown, which strongly suggests that PSAP is playing an important role in α-synuclein regulation. We also show that mRNA transcription of the *SNCA* gene is not affected by PSAP overexpression, which indicates that the regulation of α-synuclein by PSAP occurs at the translational or post-translational level, rather than the transcriptional level. Furthermore, we revealed that PSAP-OE cells showed altered autophagy, recapitulating the involvement of PSAP in autophagy-lysosomal function (Motta et al. 2016). The association between lysosomal storage disease and autophagy deficits has been shown in many studies (Ward et al. 2016; Seranova et al. 2017). It was revealed that lack of saposin C triggers enhanced autophagy, which results from reduced levels and enzymatic activities of cathepsin B and cathepsin D (CTSD) (Tatti et al. 2012). CTSD is an endopeptidase that cleaves PSAP into four saposins (Hiraiwa et al. 1997a). Aside from its property to cleave PSAP, CTSD is also responsible for α-synuclein degradation (Sevlever et al. 2008). Recently, it was proposed that the network of three lysosomal proteins, CTSD, PSAP, and progranulin, is associated with α-synuclein clearance (Tayebi et al. 2020). Based on our findings and literature, it is possible that PSAP may enhance α-synuclein degradation via interaction with CTSD. Further investigation of the connection between the network proteins and α-synuclein degradation may provide us with a key to understanding α-synuclein pathology in PD patients.

According to the fact that mutations in the *GBA1* gene are the common risk factors for PD, and saposin C is an activator of GCase, the link between GCase with saposin C in PD has been explored (Yap et al. 2013; Ouled Amar Bencheikh et al. 2018). Some studies reported that glucosylceramide, a substrate of GCase, is important for lipid raft formation and serves as a site for α-synuclein aggregation (Mazzulli et al. 2016; Taguchi et al. 2017; Zunke et al. 2018). In addition, it was demonstrated that saposin C binds to GCase competitively with α-synuclein both in solution and on the lipid vesicle, which protects GCase from α-synuclein inhibition (Yap et al. 2013). Based on these observations, we investigated the interaction between α-synuclein, saposin C, and glucosylceramide by generating an intra-lysosomal environment in vitro. Our results show that saposin C promotes the dissociation of α-synuclein from glucosylceramide-rich lipid vesicles in acidic conditions. This indicates that saposin C may prevent α-synuclein aggregation or accumulation by detaching it from the lipid membranes in the lysosome, which leads to efficient α-synuclein clearance. Thus, we suggest that the observed decrease in α-synuclein levels in PSAP-OE cells is attributed to the ability of saposin C to counteract α-synuclein forming aggregations on the lysosomal membrane and promotes efficient removal of α-synuclein.

However, since we overexpressed or knocked down the whole PSAP in the cells, there is a possibility that other saposins may also contribute to decreasing α-synuclein levels. For example, a recent study revealed the association between saposin D mutation and PD (Oji et al. 2020). Aggregation of α-synuclein was observed in skin fibroblast and induced-pluripotent stem cell–derived dopaminergic neurons from saposin D mutant PD patients (Oji et al. 2020). Therefore, the specific roles of saposin C in PD pathology require further investigation. One opportunity to explore saposin C roles is to generate saposin C–specific knockout cells by introducing mutations in the saposin C domain of PSAP (Vaccaro et al. 2010).

A limitation of this study may be that we decided to use undifferentiated SH-SY5Y cells to study the neuroprotective roles of PSAP and saposin C. Although differentiated cells are considered a better model for PD, it has been reported that most of the genes belonging to the major PD pathways and modules are intact in an undifferentiated SH-SY5Y cell line (Krishna et al. 2014). We, therefore, believe that our results provide further evidence that increased levels of PSAP, and particularly saposin C, may counteract α-synuclein accumulation and that strategies to elevate PSAP and saposin C may be beneficial against PD.

## Conclusion

Our results conclude that altered levels of PSAP, particularly saposin C, could underlie pathophysiological events of PD. Our findings propose that increasing PSAP levels may counteract α-synuclein pathology by enhancing α-synuclein clearance, which contributes to preventing the development of PD.

## Supplementary Information

Below is the link to the electronic supplementary material.

Supplementary file1 (TIF 1593 KB)

Supplementary file2 (TIF 2863 KB)

Supplementary file3 (TIF 1121 KB)

Supplementary file4 (TIF 970 KB)

## Data Availability

The datasets and materials used and/or analyzed during the current study are available from the corresponding author on reasonable request.
