# Supplementary figures and images for: Prosaposin Reduces α-Synuclein in Cells and Saposin C Dislodges it from Glucosylceramide-enriched Lipid Membranes
Source: J Mol Neurosci. 2022 Sep 24;72(11):2313–25. doi: 10.1007/s12031-022-02066-y (PMC9726671; doi:10.1007/s12031-022-02066-y)

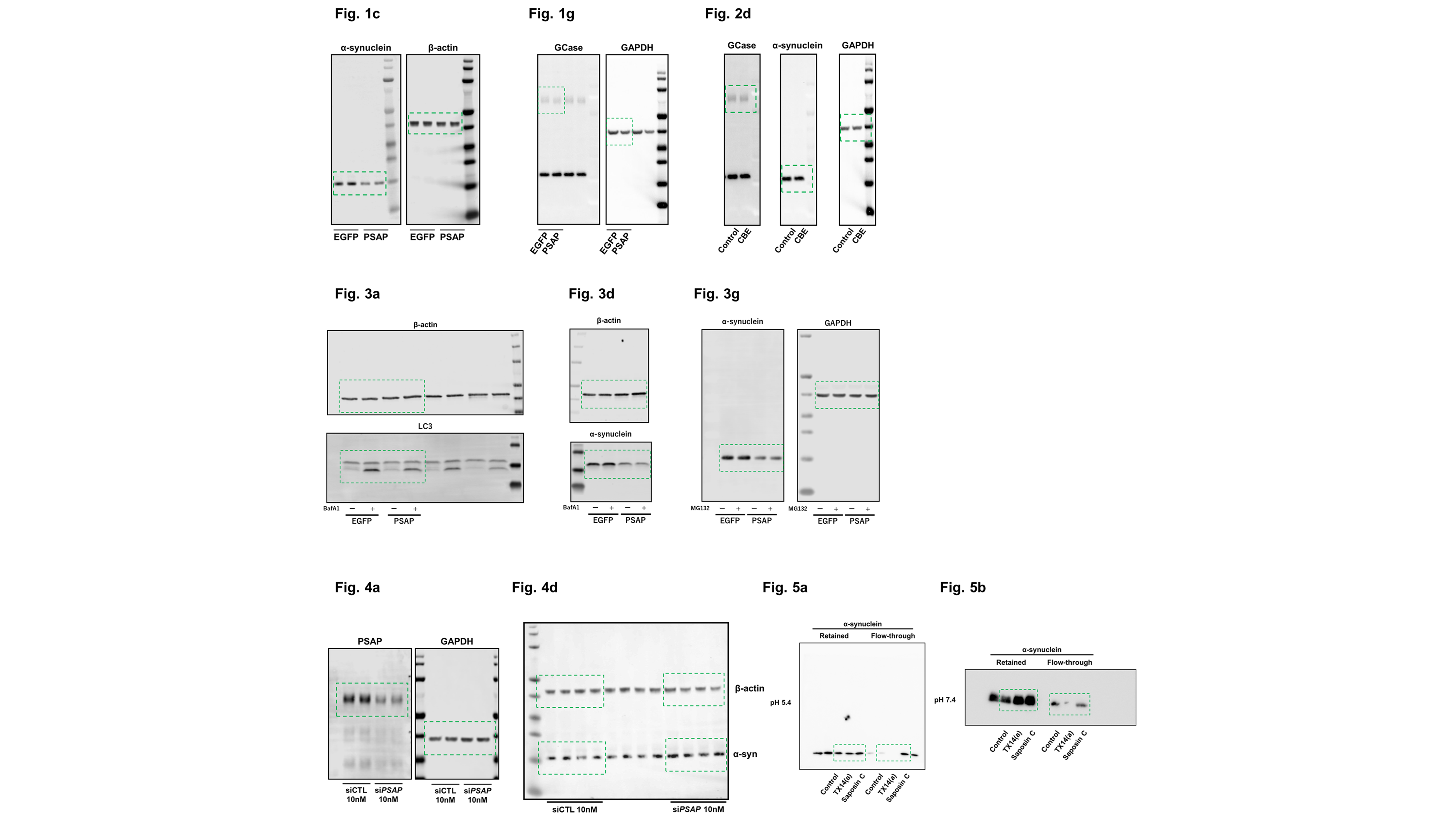

Supplement: Supplementary file 1 — Supplementary file1 (TIF 1593 KB) [file 12031_2022_2066_MOESM1_ESM.tif]

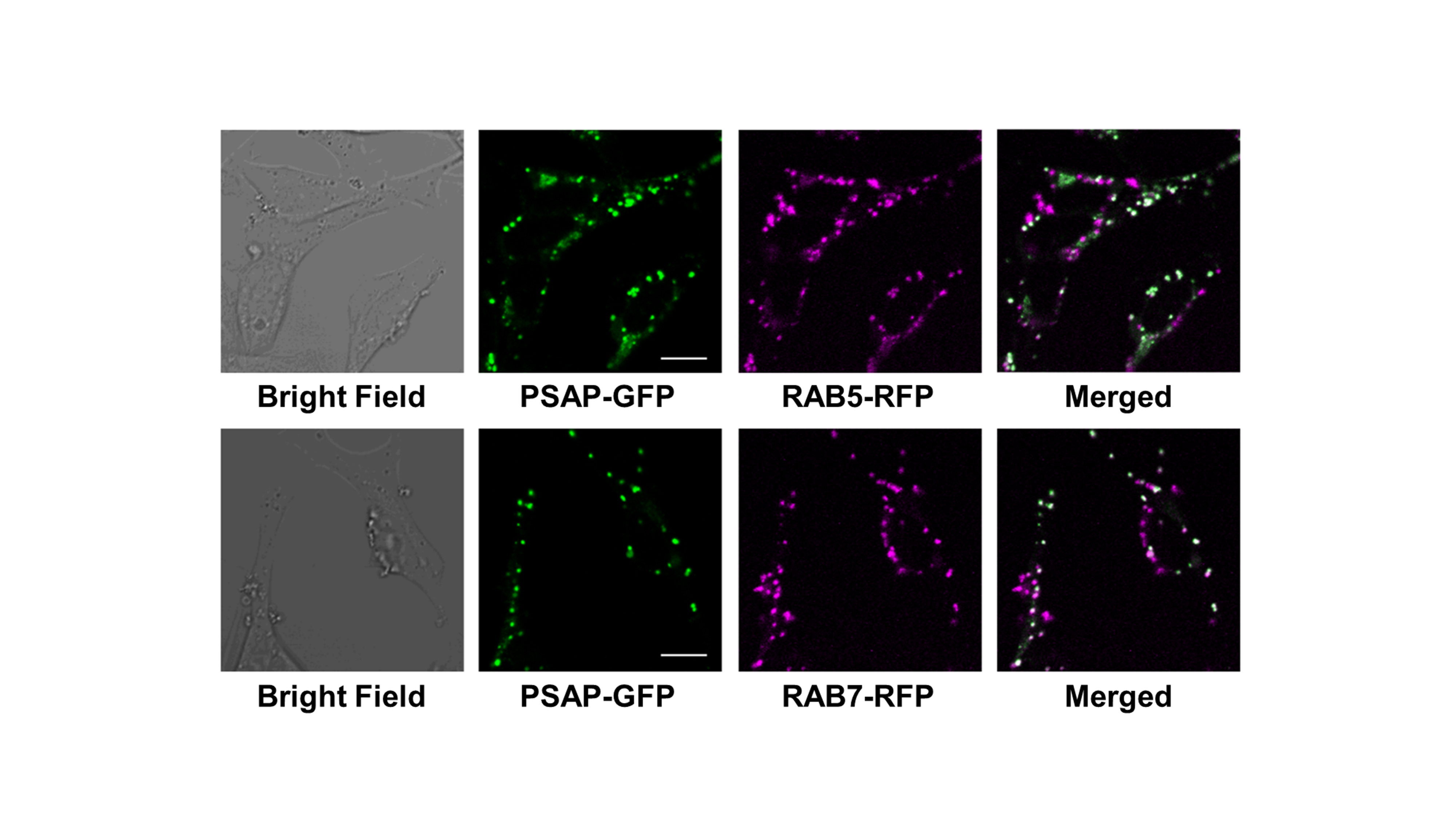

Supplement: Supplementary file 2 — Supplementary file2 (TIF 2863 KB) [file 12031_2022_2066_MOESM2_ESM.tif]

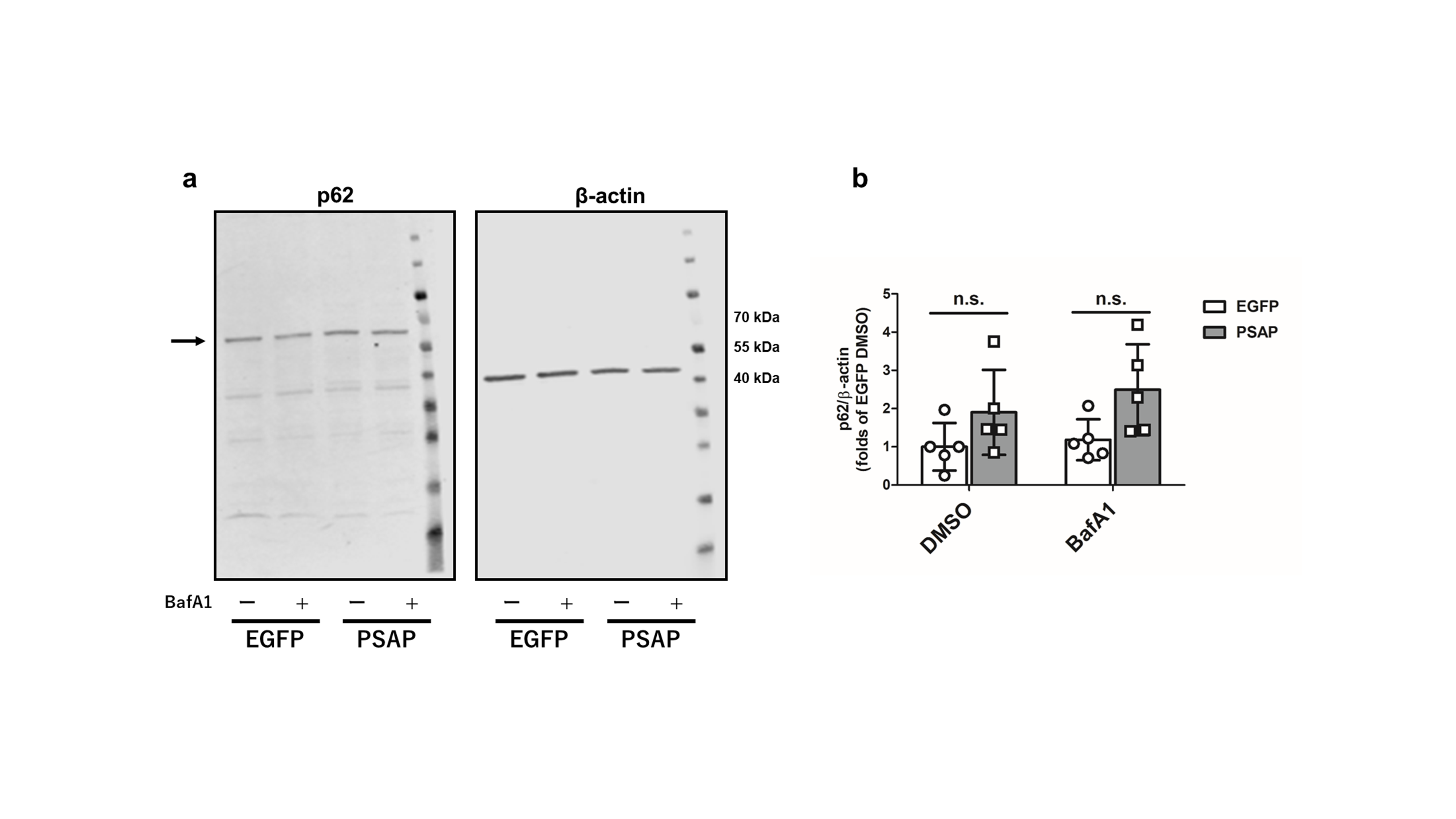

Supplement: Supplementary file 3 — Supplementary file3 (TIF 1121 KB) [file 12031_2022_2066_MOESM3_ESM.tif]

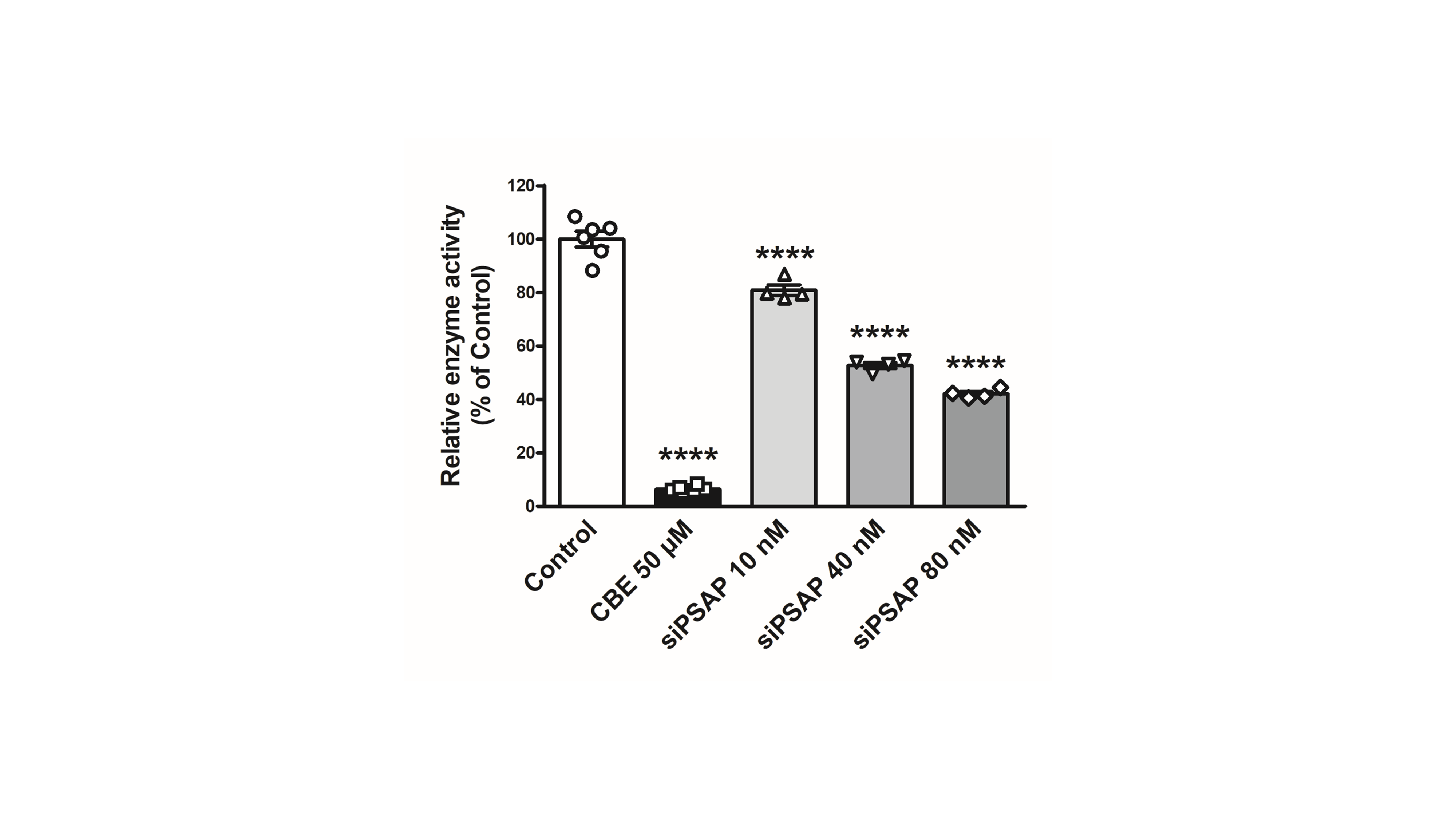

Supplement: Supplementary file 4 — Supplementary file4 (TIF 970 KB) [file 12031_2022_2066_MOESM4_ESM.tif]
